# Plasma Treatment of Poly(ethylene terephthalate) Films and Chitosan Deposition: DC- vs. AC-Discharge

**DOI:** 10.3390/ma13030508

**Published:** 2020-01-21

**Authors:** Tatiana S. Demina, Mikhail S. Piskarev, Olga A. Romanova, Andrey K. Gatin, Boris R. Senatulin, Elena A. Skryleva, Tatiana M. Zharikova, Alla B. Gilman, Alexander A. Kuznetsov, Tatiana A. Akopova, Peter S. Timashev

**Affiliations:** 1 Enikolopov Institute of Synthetic Polymeric Materials, Russian Academy of Sciences, 70 Profsoyuznaya str., Moscow 117393, Russia; mikhailpiskarev@gmail.com (M.S.P.); plasma@ispm.ru (A.B.G.); kuznets24@yandex.ru (A.A.K.); akopova@ispm.ru (T.A.A.); 2 Institute for Regenerative Medicine, Sechenov First Moscow State Medical University (Sechenov University), 8-2 Trubetskaya str., Moscow 119991, Russia; zharikova.tm@gmail.com (T.M.Z.); timashev.peter@gmail.com (P.S.T.); 3 NRC Kurchatov Institute, 1 Akademika Kurchatova pl., Moscow 123182, Russia; olga.romanova034@gmail.com; 4 Semenov Institute of Chemical Physics, Russian Academy of Sciences, 4/1 Kosygina str., Moscow 119991, Russia; akgatin@yandex.ru; 5 National University of Science and Technology “MISiS”, 4 Leninskiy pr., Moscow 119049, Russia; borisrs@yandex.ru (B.R.S.); easkryleva@gmail.com (E.A.S.); 6 Research Institute for Uronephrology and Reproductive Health, Sechenov First Moscow State Medical University (Sechenov University), 8-2 Trubetskaya str., Moscow 119991, Russia; 7 Institute of Photonic Technologies, Research center “Crystallography and Photonics”, Russian Academy of Sciences, 2 Pionerskaya str., Troitsk, Moscow 142190, Russia

**Keywords:** plasma treatment, poly(ethylene terephthalate), chitosan, cell adhesion, surface modification

## Abstract

Plasma treatment is one of the most promising tools to control surface properties of materials tailored for biomedical application. Among a variety of processing conditions, such as the nature of the working gas and time of treatment, discharge type is rarely studied, because it is mainly fixed by equipment used. This study aimed to investigate the effect of discharge type (direct vs. alternated current) using air as the working gas on plasma treatment of poly(ethylene terephthalate) films, in terms of their surface chemical structure, morphology and properties using X-ray photoelectron spectroscopy, scanning electron microscopy, atomic force microscopy and contact angle measurements. The effect of the observed changes in terms of subsequent chitosan immobilization on plasma-treated films was also evaluated. The ability of native, plasma-treated and chitosan-coated films to support adhesion and growth of mesenchymal stem cells was studied to determine the practicability of this approach for the biomedical application of poly(ethylene terephthalate) films.

## 1. Introduction

Poly(ethylene terephthalate) (PET), a thermoplastic polyester, is one of the most widely used polymers in industry. In spite of its non-biodegradability, PET has a long successful history of application in biology and medicine [1,2]. Particularly, PET inserts or membranes are widely proposed as permeable supports for the cultivation of both anchorage-depended and anchorage-independent cells. To improve cell-polymer interaction, surface functionalization of these supports via collagen or fibronectin coating is used. The main forms of PET-based materials used in clinical application are woven or knitted structures for ligament reconstruction, aortic anastomoses, etc. Surface modification of these PET materials could also be tailored in terms of cell-polymer or blood-polymer interaction as a function of future application [3]. Plasma treatment could be used as a direct approach to control these interactions or as a tool to create reactive sites for immobilization/loading of bioactive components, including anticoagulant and antithrombotic agents [4].

Among a number of methods of surface modification, plasma treatment is a well-known and highly effective technique to achieve the required surface properties without application of additional chemical reagents, which is especially desirable for modification of materials being developed for biomedical application [5,6,7,8]. Another significant benefit of plasma treatment relies on the possibility to vary the material’s surface characteristics, namely chemical structure, morphology, hydrophilicity and surface charge by changing a number of plasma-processing parameters, i.e., discharge type, duration and nature of working gas [9,10,11,12]. The majority of these processing conditions are easily adapted, but discharge type is mainly determined by equipment and, thus, should be carefully chosen in advance. Discharge type determines mainly the nature of the active plasma components (electrons and ions) affecting the treated surface. For example, an alternating current (AC) discharge, widely used for laboratory experiments, led to a changing of the electrode’s charge sign during the plasma treatment process, thus causing a mixing of both components and their simultaneous influence on the treated surface. On the other hand, direct current (DC) discharge led to a separation of the active plasma components between two electrodes; therefore, surface modification proceeds as a function of the electrode used for the treatment. Therefore, the results of plasma treatment, such as etching, changes of the chemical structure and accordingly the properties of the surface layer, would be significantly varied as a function of discharge type. All these surface parameters are of great importance for further cell-material interaction.

Plasma treatment could also be used as a tool for surface activation prior to immobilization or grafting of bioactive components on the materials. Thus, it was previously shown to be effective for subsequent immobilization of various bioactive components [13,14,15,16,17,18]. Chitosan is one of the widely used natural polymers for immobilization, because of its biocompatibility, biodegradability, antibacterial activity and its ability to act as a site for binding of other bioactive components or cell attachment [19]. There are a number of works describing various approaches to chitosan immobilization or chitosan-based coating deposition onto hydrophobic polymeric materials [20,21,22,23]. Some of these approaches could be more effective than plasma treatment, but require the application of specific and rather expensive techniques or additional components, which makes plasma treatment the easiest way to achieve surface modification and chitosan immobilization.

This study aimed to evaluate the effectiveness of plasma treatment as a direct tool of PET surface modification or as an approach for surface functionalization prior to immobilization of chitosan onto plasma-treated films. The effect of discharge type, i.e., DC-discharge (at the cathode or at the anode) or AC-discharge was evaluated in terms of the changes in surface chemical structure, morphology and properties as well as an ability to regulate the adhesion and growth of animal cells.

## 2. Materials and Methods

The samples of commercially available poly(ethylene terephthalate) (PET) films (40 μm, PETLAIN BT 1010 E, Superfilm, Turkey) were cleaned in ethanol and dried at RT before use. Low-molecular-weight chitosan (60 kDa) with degree of acetylation of 0.1 was prepared by the solid-state synthesis in Institute of Synthetic Polymeric Materials of Russian Academy of Sciences as reported earlier [24]. All solvents were of analytical grade and were used as received.

The plasma treatment of AC-discharge was carried out using CUTE-1MPR (Femto Science Inc., Gyeonggi, Korea) operating at 40 kHz. The chamber (140 × 200 × 110 mm; electrode area of ~200 cm^2^) was evacuated to a base pressure of 20 mTorr before the working gas, synthetic air, was introduced at a flow rate of 20 sccm. Subsequently, the glow discharge was ignited at 50 W within 10–60 s. DC-discharge (50 mA) plasma treatment was carried out using a laboratory-made setup consisting of two electrodes of 18 cm in diameter (electrode area of 254 cm^2^) with a gap of 5 cm [25]. Plasma treatment was realized at the anode or at the cathode using filtered air as working gas (~20 Pa) within 10–60 s, as previously described [26]. The setups and conditions of AC- and DC-discharge plasma treatment were kept as similar as possible, but optimal for treatment using both discharge types. The size of PET films was 1.5 × 2.5 cm (i.e., 3.75 cm^2^) to provide a stable ignition and sample surface treatment during DC-discharge plasma modification. The homogeneity of the DC-discharge surface modification was confirmed by measurements of contact angles of wettability on different spots of the treated film (from one edge to another). Since the effect of plasma treatment has a tendency to diminish in time, all future experiments were carried out within 24 h after the treatment.

Chitosan was dissolved in 1% CH_3_COOH over 2 h to achieve 1 wt% concentration. Then, PET films immediately after plasma treatment were immersed into the chitosan solutions for 2 h at 37 °C. Afterwards, they were carefully washed with distilled water and dried in a dust-free chamber at RT.

The surface properties of native PET films as well as chitosan-coated films were characterized by contact angle (θ) measurements with an Easy Drop DSA100 (KRUSS, Germany) using two test liquids (bidistilled water (mQ) and glycerol) within 1 min after plasma treatment. The values of work of adhesion (W_a_), the total surface energy (γ), and its polar (γ^р^) and dispersion (γ^d^) components were calculated according to standard equations [27]. The measurements of contact angles were carried out at least in triplicate and presented as a mean value (±1°) for each experimental point.

The chemical structure of the initial and plasma-treated PET films was studied by X-ray photoelectron spectroscopy (XPS) with an aim of PHI 5500 VersaProbeII spectrometer. The experiments were carried out in an ultrahigh vacuum of 5 × 10^–8^ Pa using monochromatic AlKα radiation (hν = 1486.6 eV, power 50 W), the diameter of the analysis area was 200 μm. The C1s and O1s binding energies were determined from the high-resolution spectra measured at a pass energy of 11.75 eV and step size of 0.1 eV. The approximation of the spectra was performed by a nonlinear least-squares method using the Gauss–Lorentz function. The calibration of the binding energy scale was adjusted using E = 284.7 eV of the C1s peak of aromatic CH groups in the spectrum [28,29].

The surface morphology of the native and plasma-treated PET films was evaluated using scanning electron (SEM) and atomic force microscopy (AFM). SEM is carried out using PhenomProX (PhenomWorld, The Netherlands) and the acquired images are given as supporting information (Figure S2). AFM was realized using Solver HV atomic-force microscope (NT-MDT, Russia) with standard HA-NC cantilevers (NT-MDT, Russia). The measurements were carried out in a tapping mode with the acquisition of surface topography and phase contrast. Root-mean-square (R_ms_) roughness values were calculated from scratch-free scan areas of 2 × 2 µm using the NOVA software version 1.1.0.1851 (NT-MDT, Zelenograd, Russia).

Fluorescein isothiocyanate (FITC) labeling was used to visualize chitosan moieties onto the plasma-treated PET films. FITC labeling was carried out by the incubation of pieces of the chitosan-coated PET films in 1mL of borate buffer containing 10 µL of 0.2 wt% FITC solution in dimethylsulfoxide at RT for 2 h. Then, the films were carefully rinsed by distilled water and observed using microscope Leica DFC7000T (Leica Microsystems, Wetzlar, Germany).

Mesenchymal stem cells (MSCs) from human adipose tissue were kindly provided by Dr E. Solovieva (National Research Centre Kurchatov Institute, Moscow, Russia).

Evaluation of the proliferation of mesenchymal stem cells on matrices was carried out using the MTT cell proliferation assay (Sigma, Cat. No. M5655, MI, USA). Cells were cultured in Dulbeccoߣs Modified Eagle Medium (DMEM) (Thermo Fisher Scientific, Cat. No:10567-014, MA, USA) with 10% fetal bovine serum, 1% PenStrep (Thermo Fisher Scientific, Cat. No: 10378016, MA, USA). The testing was performed on days 1 and 2 after seeding 8 × 10^3^ cells/cm^2^ (three replicates for each time point) on 0.25 cm^2^ slides. The MTT metabolite absorbance was measured using VICTOR X3 Microplate Reader (PerkinElmer, Shelton, CT, USA).

For viability analysis fluorescent viability/cytotoxicity Kit (Invitrogen, Cat. No. L-3224, Camarillo, USA) was used according to the manufacturer’s protocol. The samples were analyzed under the fluorescence microscope Zeiss Axiovert 40 CFL (Jena, Germany).

## 3. Results and Discussion

### 3.1. Plasma Treatment of PET Films

**Figure 1:**
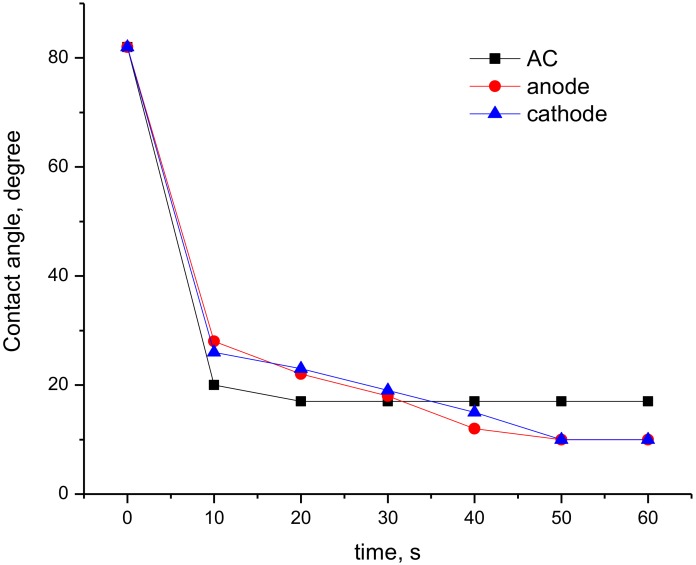
Dependence of contact angle of wettability as a function of treatment time using AC- and DC-discharge. Curves for DC-treated films are adapted by permission from Springer Nature: Springer Nature, High Energy Chem., Piskarev MS, Gilman AB, Gatin AK, Gaidar AI, Kurkin TS, Kuznetsov AA, Copyright (2019).

Since plasma treatment is widely used as a tool to increase surface hydrophilicity, wettability contact angle measurements were carried out. It was revealed that plasma treatment of the PET films led to a significant increase in surface hydrophilicity irrespective of the discharge type. The contact angle of wettability by water (θ_w_) drops from 80° (non-treated PET films) to 17° for AC-plasma or to almost full water drop spreading (10–12°) in the case of DC-discharge plasma treatment. However, the decrease of θ_w_ as a function of treatment time depended significantly on discharge type (Figure 1). The main changes in θ_w_ proceeds in the first 20 s of plasma treatment using AC-discharge, while prolonged treatment at these conditions did not affect wettability. In contrast, DC-discharge needs at least 50 s to achieve this equilibrium in wettability. The difference in effectiveness of DC-discharge at the anode or at the cathode in terms of surface hydrophilization was insignificant. To eliminate the time-dependence effect, all further studies were carried out using PET samples treated for 60 s using both types of discharge.

Calculation of values of work of adhesion, the total surface energy and its components for PET films treated for 60 s showed no significant difference as a function of discharge type (Table 1). Plasma treatment led to a ~4.4-fold increase in the polar component of surface energy, whereas the dispersion component of the surface energy increased by only ~12%. Because the polar component is particularly sensitive to the presence of polar groups in the surface layer, a change in the chemical composition could be the most likely reason of this effect.

A study of the chemical structure of the surface layer of initial and plasma-treated PET films was carried out using XPS. According to the atomic composition data in Table 2, plasma treatment caused the oxidation of the surface layer in all cases, except for DC-discharge plasma treatment at the anode. Plasma treatment of PET films in all studied cases led also to the formation of small amounts of nitrogen-containing groups, which is logical taking into account the nature of the working gas (air). The presence of aluminum within the surface layer of DC-treated samples could be explained by the etching of electrodes and the subsequent deposition of aluminum oxides onto the PET surface, which is a common process during DC-discharge plasma treatment. To reveal details in surface chemical composition changes as a function of discharge type, a study of C1s and O1s core-level spectra was carried out.

Deconvolution data of high-resolution XPS spectra of initial and plasma-treated samples are shown in Table 3, while C1s and O1s spectra can be found in Figure S1 in the supporting information. Initial C1s spectra of PET could be deconvoluted into three main peaks and a weak peak at 290–292 eV, which could be linked to shake-up satellite of the PET aromatic groups. The main peaks in C1s spectra of PET could be assigned to C−C/C−H of aromatic ring at 284.7 eV; C−O at 286.3 eV; O=C–O at 288.7 eV. The deconvolution of O1s spectra confirms the presence of these oxygen-containing groups—O=C–O at 531.6 eV and C–O at 533.2 eV.

Changes in the C1s and O1s core-level spectra after plasma treatment strongly depended on discharge type and, therefore, on active plasma components affecting the surface layer. Treatment of PET by AC-plasma and DC-discharge at the cathode led to surface oxidation due to ion bombardment. As could be seen in Table 3 the main increase in oxygen-containing groups under these plasma treatment conditions was caused by formation of C−O bonds. On the contrary, treatment at the anode, i.e., by electrons mainly, caused the formation of a surface layer containing the lowest amount of oxygen-containing groups. Apparently, electrons could easily cleavage C–C bonds, thus, leading to macromolecules cross-linking. An absence of the peak at 290–292 eV linked to the aromatic cycle supports the conclusion that the surface layer of anode-treated sample consists of cross-linked fragments of PET macromolecules. Analysis of O1s core-level spectra deconvolution of the films treated by DC-discharge plasma at the anode or at the cathode showed an opposite contribution of C−O and O=C–O groups as a function of treatment electrode used. Treated at the anode PET films possessed highest amount of carboxyl groups in the surface layer. Thus, in terms of changes of surface chemical structure the AC-discharge provided the mildest conditions.

**Figure 2:**
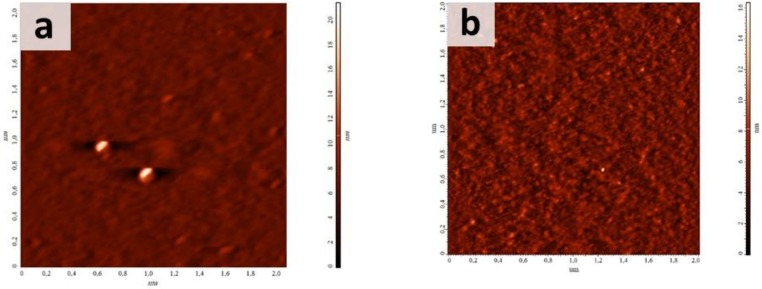

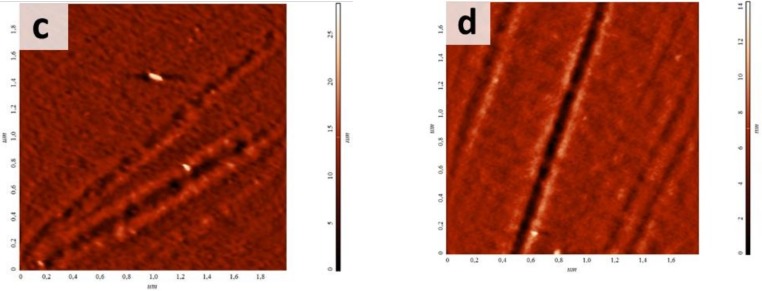
Atomic force microscopy (AFM) images of (**a**) initial and plasma-treated PET films using (**b**) AC- and DC-discharge (**c**) at the cathode and (**d**) at the anode.

Another process taking place during a plasma treatment is surface etching, which also depends on active plasma components affecting treated surface. As could be seen from AFM micrographs presented in Figure 2, the initial PET film possessed a smooth surface (R_ms_ of 0.9 nm). Plasma treatment in all cases led to an increase of R_ms_ up to 1.2 nm, 1.7 nm and 1.3 nm for the films treated using AC-discharge or DC-discharge at the cathode and at the anode, respectively. However, AC-discharge plasma treatment caused a uniform surface etching and an increase of R_ms_ up to 1.2 nm, while DC-discharge led to the appearance of strips, which were more pronounced on anode-treated films. We suppose that these strips are related to oriented domains forming during the polymer stretching in the process of the film fabrication. High orders of macromolecules within these domains made them more stable under plasma action. PET treatment at the anode led to a more significant polymer destruction and a higher level of etching of preferably unoriented domains. SEM micrographs of non-treated and plasma-treated films show these pronounced on the anode-treated sample rather than on the cathode-treated one (see Figure S2 in supporting information).

The observed changes in surface chemical structure and morphology are enough to modify surface properties of PET films, such as wettability (see Table 1), cell adhesion and growth (see Section 3.1). However, it is well known that the effect of plasma treatment diminishes over time (the so-called ageing process) leading to restoration (usually, only a partial one) of the initial surface properties due to a hydrophobic recovery, i.e., re-arrangement of polar groups generated on the surface to the bulk phase of the polymer [30,31,32]. This issue could limit the use of the plasma-treated films for biomedical purposes.

**Figure 3:**
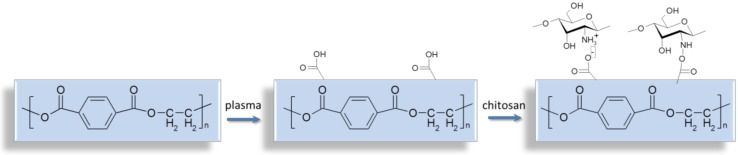
Schema of PET film plasma treatment and chitosan immobilization.

In the frame of the present study, we evaluated the effectiveness of DC- and AC-discharge plasma treatment not only as a direct approach to surface modification, but also as a tool for surface activation prior to the immobilization of chitosan. As it was shown using XPS, the plasma treatment of PET films led to the generation of polar oxygen-containing groups, which could interact with chitosan amino groups (see Figure 3). Thus, the plasma treatment of PET films could enhance the adsorption of chitosan from its solution. In the frame of our research, the films after incubation in chitosan solution were thoroughly rinsed to eliminate “free” polysaccharide. To confirm the presence of chitosan on the plasma-treated PET surfaces, these samples were labeled with FITC, a fluorescein reactive to amino groups, and observed using fluorescent microscopy. All chitosan-coated films demonstrated emission coming from FITC bonded to the chitosan-coated films (see Figure S3 in supporting information section). We suppose, that chitosan could be linked to plasma-treated PET films via ionic or covalent bonds.

The effect of chitosan immobilization on PET films’ wettability was evaluated as well. As could be seen from Table 1, immobilization of chitosan onto non-treated PET film did not led to significant changes in contact angle, surface energy and polar and dispersion components. On the contrary, the immobilization of chitosan on plasma-treated films led to significant changes of contact angles in contrast to native and freshly plasma-treated samples. The value of θ_w_ increased from 10–17° found for freshly treated films, to 49–54°. Immobilization of chitosan led to a ~1.5-fold decrease in surface energy in comparison with plasma-treated films due to the decrease of its polar component. Coating DC-discharge plasma-treated films with chitosan caused significant a decrease of the dispersive component of the surface energy, while AC-discharge treated films showed a significant increase of γ^d^ of surface energy components. As can be seen from SEM micrographs presented in Figure S2 in the supporting information, chitosan immobilization didn’t lead to significant changes in surface morphology of non-treated and plasma-treated films.

### 3.2. Cell Adhesion and Growth

**Figure 4:**
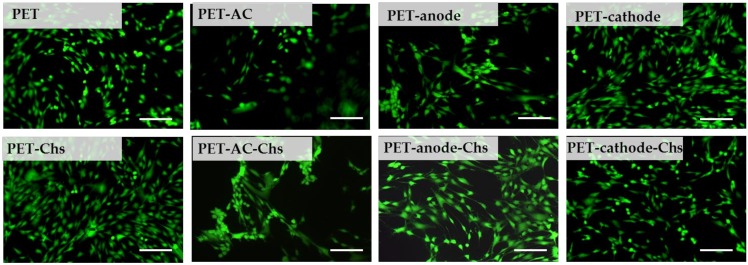
Morphology of human mesenchymal stem cells on initial and plasma-treated PET films as well as ones coated with chitosan (48 h in culture). Scale bar is 100 μm.

The effect of plasma treatment and further immobilization of chitosan was evaluated in terms of the adhesion and growth of human mesenchymal stem cells. As can be seen in Figure 4, DC-discharge plasma treatment didn’t lead to significant changes in cell morphology, while cells cultured on AC-treated films possessed a round shape. Cell adhesion and proliferation onto PET films after chitosan immobilization depend on the type of substrate pre-treatment before coating. Thus, the cells cultured onto chitosan-coated AC-treated films showed the same morphology as ones on AC-treated films without chitosan coating. According to MTT-assay, the proliferation activity of cells cultured on AC-discharge-treated films was the lowest (see Figure S4 in supporting information). In any case, the cells were successfully proliferated on the all studied samples and reached confluency in 48 h. However, the formed cell monolayer was easily detached irrespective of any plasma treatment and chitosan coating.

Thus, plasma treatment allows us to regulate the PET film’s surface chemical structure, morphology and properties as a function of discharge type, as well as to activate it for chitosan immobilization. Although plasma treatment could affect surface properties, low cell adhesion on PET substrates was observed in the long run.

## 4. Conclusions

Plasma treatment by DC- or AC-discharge led to a drastic increase of the hydrophilicity of PET films. DC-discharge treatment allowed us to decrease the contact angle of wettability from 80° to full spreading, while AC-discharge to only 17°. The contact angle change was faster in the case of AC-discharge. The formation of nitrogen-containing function groups was observed in all cases, while the increase of the amount of oxygen-containing ones was determined in the case of AC-discharge and DC-discharge at the cathode only. As a function of the electrode used during DC-discharge plasma treatment, the contribution of C−O and O=C–O groups to the oxygen content differed. DC-discharge appeared to be harsher in terms either of the increase of surface roughness or the formation/change of nitrogen/oxygen functional groups. The formed specific characteristics of plasma-treated films affect adsorption of chitosan onto PET films as well as the ability to support the adhesion and growth of mesenchymal stem cells. It should be specially noted that the effect of plasma-treatment conditions was more pronounced for cell adhesion and viability than the presence of chitosan on the PET surface. However, irrespective of plasma treatment and chitosan coating, the formed cell monolayer was easily detached from PET substrates.

## Figures and Tables

**Table 1:** Surface properties of the Poly(ethylene terephthalate) (PET) films treated by AC or DC plasma for 60 s as a function of discharge type.

Sample	θ, deg		W, mJ/m^2^		Surface Energy, mJ/m^2^		
	θ_w_	θ_glyc_	water	glycerol	γ	γ^p^	γ^d^
PET	80 ± 1	73 ± 1	85.4 ± 1.3	81.9 ± 1.1	26.5 ± 0.7	12.4 ± 0.5	14.1 ± 0.2
PET-AC	17 ± 1	15 ± 1	142.4 ± 0.4	124.6 ± 0.3	70.0 ± 0.4	53.5 ± 0.4	16.5 ± 0.1
PET-cathode	10 ± 1	9 ± 1	144.5 ± 0.2	126.0 ± 0.2	72.2 ± 0.2	55.8 ± 0.2	16.4 ± 0.1
PET-anode	12 ± 1	10 ± 1	144.0 ± 0.3	125.8 ± 0.2	71.6 ± 0.3	55.0 ± 0.3	16.6 ± 0.1
PET-Chs	80 ± 1	73 ± 1	85.4 ± 1.3	81.9 ± 1.1	26.5 ± 0.7	12.4 ± 0.5	14.1 ± 0.2
PET-AC-Chs	49 ± 1	45 ± 1	120.6 ± 1.0	108.2 ± 0.8	49.9 ± 0.8	15.6 ± 0.1	34.4 ± 0.7
PET-cathode-Chs	50 ± 1	57 ± 1	119.6 ± 1.0	97.9 ± 0.9	53.0 ± 0.8	48.3 ± 0.6	4.7 ± 0.2
PET-anode-Chs	54 ± 1	56 ± 1	115.6 ± 1.0	98.9 ± 0.9	46.9 ± 0.8	38.6 ± 0.6	8.3 ± 0.2

**Table 2:** Surface chemical structure of the initial and the plasma-treated (60 s) PET films.

Sample	Atomic Concentration, %				
	С	O	N	Al	O/C
PET	73.0	27.0	−	−	0.37
PET-AC	66.0	31.3	2.5	−	0.47
PET-cathode	59.9	34.9	1.2	4.0	0.58
PET-anode	70.8	25.6	1.8	1.8	0.36

**Table 3:** Deconvolution of X-ray photoelectron spectroscopy (XPS) C1s and O1s core-level spectra of initial and plasma-treated PET films.

Sample	Atom Level	Peak Energy, eV	Atomic, %	Bond
PET	C1s	284.7	63	C−C/C−H
		286.3	20	C−O
		288.7	17	O=C–O
	O1s	531.7	43	O=C
		533.3	57	С–О
PET-AC	C1s	284.7	56	C−C/C−H
		286.4	25	C−O
		288.7	19	O=C–O
	O1s	531.5	40	O=C
		533.0	60	С–О
PET-cathode	C1s	284.7	56	C−C/C−H
		286.3	26	C−O
		288.7	18	O=C–O
	O1s	531.7	33	O=C
		533.2	67	С–О
PET-anode	C1s	284.7	65	C−C/C−H
		286.3	22	C−O
		288.7	13	O=C–O
	O1s	532.0	67	O=C
		533.3	33	С–О

## Supplementary Materials

The following are available online at https://www.mdpi.com/1996-1944/13/3/508/s1, Figure S1: C1s and O1s spectra of plasma-treated PET films, Figure S2: SEM micrographs of non-treated PET film and chitosan-coated plasma-treated PET films, Figure S3: Fluorescent microscopy of FITC-labeled initial and plasma-treated PET films after immobilization of chitosan, Figure S4: MTT-proliferation assay results (OD value) for human mesenchymal stem cells onto initial and plasma-treated PET films with/without chitosan-coating (24 and 48 h in culture).
