# Supplementary material for: Plasma Treatment of Poly(ethylene terephthalate) Films and Chitosan Deposition: DC- vs. AC-Discharge
Source: Materials (Basel). 2020 Jan 21;13(3):508. doi: 10.3390/ma13030508 (PMC7040612; doi:10.3390/ma13030508)
Supplement: Supplementary file 1 [file materials-13-00508-s001.pdf]

materials

MDPI

Article

Plasma Treatment of Poly(ethylene terephthalate) Films and Chitosan Deposition: DC– vs. AC–Discharge

Tatiana S. Demina[1,2,*], Mikhail S. Piskarev [1], Olga A. Romanova [3], Andrey K. Gatin [4], Boris R. Senatulin [5], Elena A. Skryleva [5], Tatiana M. Zharikova [2], Alla B. Gilman [1], Alexander A. Kuznetsov [1], Tatiana A. Akopova [1] and Peter S. Timashev [2,4,6]

1 Enikolopov Institute of Synthetic Polymer Materials, Russian Academy of Sciences, 70 Profsoyuznaya str., 117393, Moscow, Russia; mikhailpiskarev@gmail.com (M.S.P.); plasma@ispm.ru (A.B.G.); kuznets24@yandex.ru (A.A.K.); akopova@ispm.ru (T.A.A.)
2 Institute for Regenerative Medicine, Sechenov First Moscow State Medical University (Sechenov University), 8-2 Trubetskaya str., Moscow, 119991, Russia; zharikova.tm@gmail.com (T.M.Z.); timashev.peter@gmail.com (P.S.T.)
3 NRC Kurchatov Institute, 1 Akademika Kurchatova pl., Moscow, 123182, Russia; olga.romanova034@gmail.com
4 Semenov Institute of Chemical Physics, Russian Academy of Sciences, 4/1 Kosygina str., Moscow, 119991, Russia; akgatin@yandex.ru
5 National University of Science and Technology "MISiS", 4 Leninskiy pr., Moscow, 119049, Russia; borisrs@yandex.ru (B.R.S.); easkryleva@gmail.com (E.A.S.)
6 Institute of Photonic Technologies, Research center "Crystallography and Photonics", Russian Academy of Sciences, 2 Pionerskaya str., Troitsk, Moscow, 142190, Russia
* Correspondence: detans@gmail.com

Received: 24 October 2019; Accepted: 16 January 2020; Published: date

	PET	PET-AC	PET-anode	PET-cathode
C1s	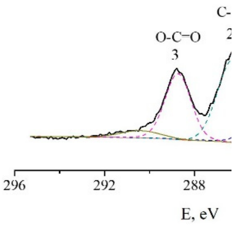	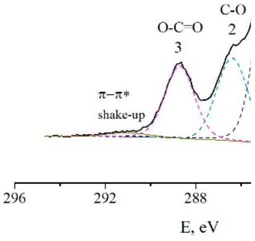	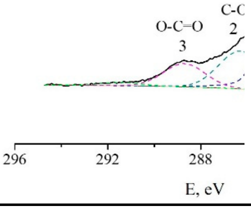	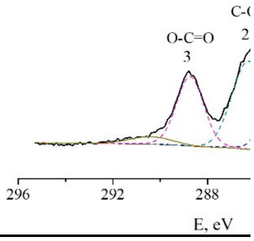

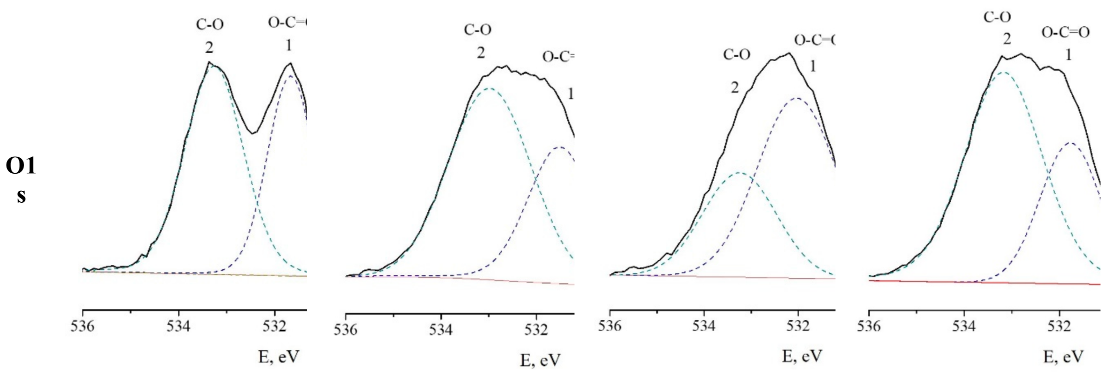

Figure S1. C1s and O1s spectra of the AC- and DC-discharge plasma treated PET films. Spectra of initial and DC-treated films are adapted by permission from Springer Nature: Springer Nature, High Energy Chem, Piskarev MS, Gilman AB, Gatin AK, Gaidar AI, Kurkin TS, Kuznetsov AA, Copyright (2019).

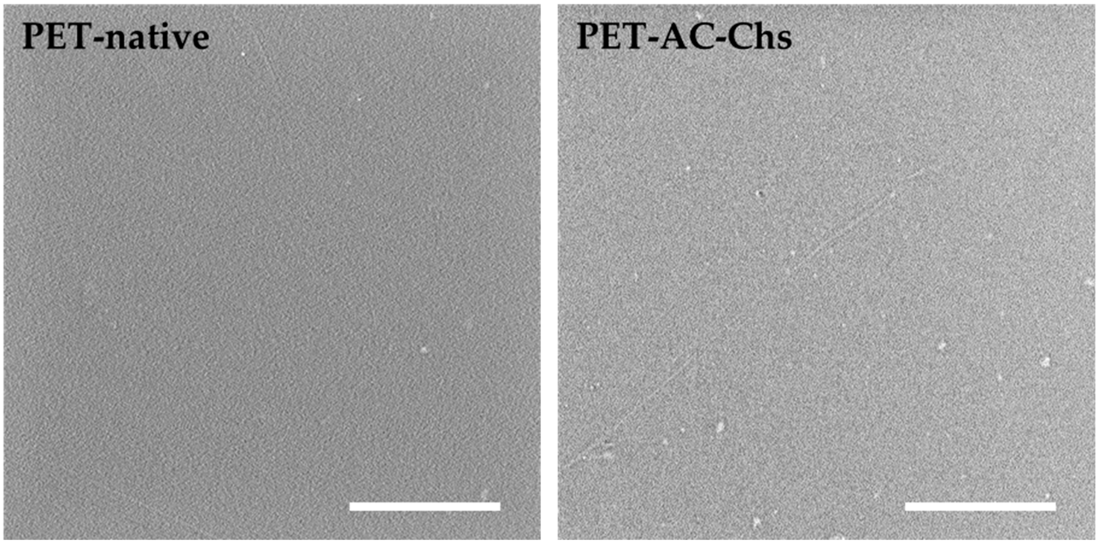

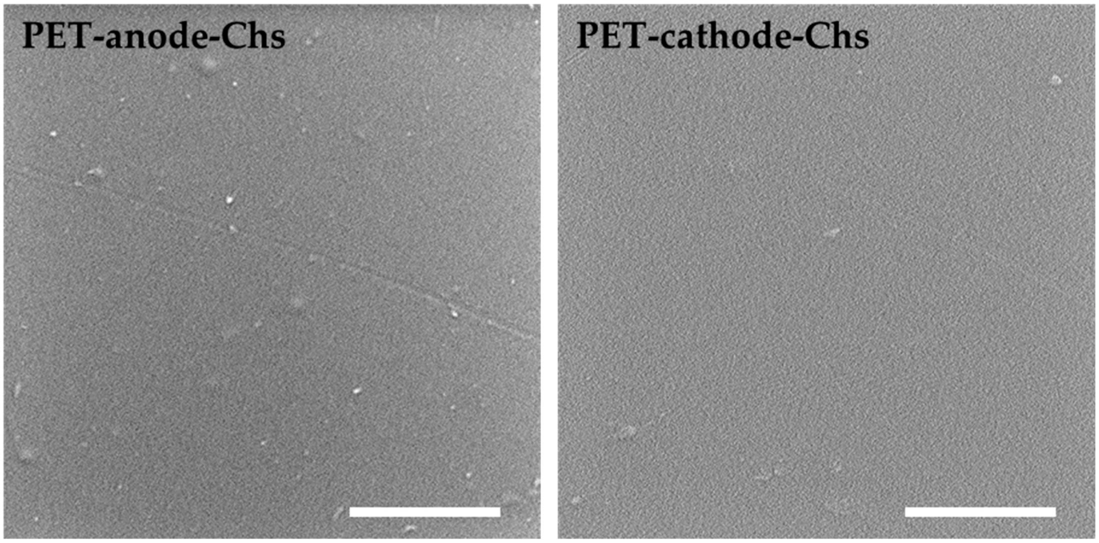

Figure S2. SEM micrographs of non-treated PET film and chitosan-coated plasma-treated PET films. Scale bar is 50 μm.

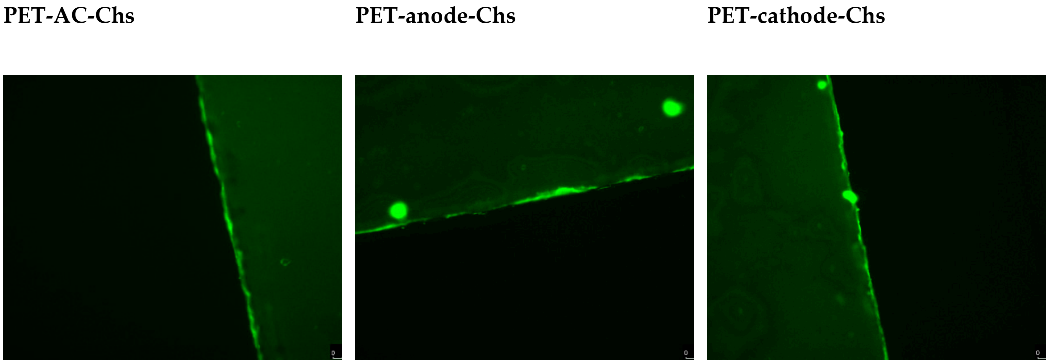

Figure S3. Fluorescent microscopy of FITC-labeled initial and plasma-treated PET films after immobilization of chitosan.

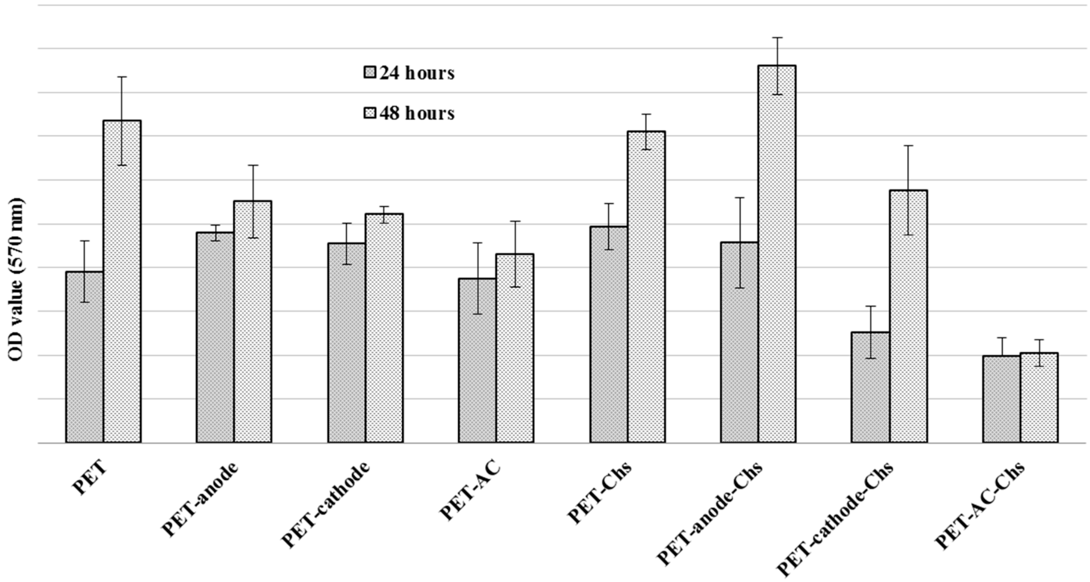

Figure S4. MTT-proliferation assay results (OD value) for human mesenchymal stem cells onto initial and plasma-treated PET films with/without chitosan-coating (24 and 48 hours in culture).

© 2020 by the authors. Submitted for possible open access publication under the terms and conditions of the Creative Commons Attribution (CC BY) license (http://creativecommons.org/licenses/by/4.0/).
